# Occupational injuries among health care workers at a public hospital in Ghana

**DOI:** 10.11604/pamj.2021.39.103.23542

**Published:** 2021-06-03

**Authors:** Helena Appiagyei, Emmanuel Kweku Nakua, Peter Donkor, Charles Mock

**Affiliations:** 1 HopeXchange Medical Center, Santasi, Kumasi, Ghana,; 2 Department of Epidemiology and Biostatistics, School of Public Health, Kwame Nkrumah University of Science and Technology, Kumasi, Ghana,; 3 Department of Surgery, Kwame Nkrumah University of Science and Technology, Kumasi, Ghana,; 4 Harborview Injury Prevention and Research Center, University of Washington, Seattle, WA, USA

**Keywords:** Africa, Ghana, health care worker, occupational injury, safety

## Abstract

**Introduction:**

information on occupational injuries to health care workers (HCWs) in Africa is limited. We sought to determine the prevalence of occupational injuries among HCWs at a Ghanaian hospital, determine the most common types of injuries, and assess HCWs' knowledge regarding occupational safety.

**Methods:**

we interviewed 246 HCWs at a government hospital regarding occupational injuries during the prior year. The sample included: nurses (77.6%), physicians (9.3%), laboratory staff (5.7%), and non-clinical staff (6.9%).

**Results:**

the 12-month prevalence of occupational injury was 29.7%. Incidence was 1.63 injuries per person-year. Leading mechanisms were needlesticks (35.4% of injuries), cuts from sharp objects (34.6%), hit by object (25.2%), and violence (24.4%). Most (62.2%) respondents had training in occupational safety. Most reported adherence to safety practices, including properly disposing sharps (86.6%) and using personal protective equipment (85.8%). However, there were gaps in knowledge. Few HCWs knew the officer in-charge for post-exposure prophylaxis (5.3%) or that there was a hospital occupational safety unit (26.4%). Many (20.8%) reported difficulty in seeking care for their injury. On multivariable analysis, correlates of injury included stress at work (aOR 2.68; 95% CI 1.26, 5.71) and being a laboratory worker (aOR 3.26; 95% CI 1.02, 10.50).

**Conclusion:**

occupational injuries to HCWs were unacceptably frequent. There is, however, a solid foundation to build on. Most HCWs had training in occupational safety and many reported adherence to safety practices. Health care workers need to be better informed of existing resources. Care for injuries needs to be improved, such as by increasing capacity for post-exposure prophylaxis.

## Introduction

Over 59 million people are engaged as health care workers (HCWs) globally [1]. The safety of these workers is a significant issue. HCWs face a variety of occupational hazards, such as exposure to blood-borne pathogens from body fluids and needlestick injuries and exposure to a variety of other pathogens such as tuberculosis. They also face hazards such as slips, trips, and violence from patients and relatives, as well as ergonomic hazards such as heavy lifting and psychosocial hazards related to shift work and stress. Needlestick or sharps injuries have been found to be the most frequent injury among HCWs in many countries, resulting in almost 16,000 hepatitis C, 66,000 hepatitis B, and 1,000 HIV infections annually worldwide [2].

Data on occupational injuries and other occupational health issues are problematic in many low- and middle-income countries (LMICs), with considerable under-reporting in official statistics. This is especially the case for sub-Saharan Africa [3]. To supplement the official statistics, there has been a modest number of research studies carried out in Africa. For example, a study of exposure to body fluids in 21 African countries [4] found a 12-month prevalence of such exposure of 66%. Gupta *et al*. documented scarcity of personal protective equipment to prevent exposure to blood-borne pathogens in 13 LMICs, including four in Africa [5]. There have also been reports on specific health issues in specific locations, such as availability of post-exposure prophylaxis for HIV in Gondar, Ethiopia [6] and prevalence of low back pain among HCWs in Sokoto, Nigeria [7].

In Ghana, several studies have focussed on hepatitis B, especially as regards the uptake and effectiveness of the vaccine [8-10]. Other studies have looked at psychological stress among nurses [11], sharps injuries among nurses [12], or health and safety for HCWs at the psychiatric hospitals in Ghana [13]. There has not been, to our knowledge, a study that estimated the incidence rate of all injuries to workers in a hospital in Ghana or evaluated the problem of safety and health for HCWs more generally in Ghana. Hence, we sought to address this deficiency and to estimate the 12-month prevalence rate and annual incidence rate of occupational injuries among HCWs at a typical government, district hospital in Ghana, and to assess the presence or absence of risk factors and safety measures.

## Methods

**Setting and study design:** Ghana is a lower-middle-income country with a population of about 30 million people [14]. Health services management is decentralized. The country operates a 3-tier health care delivery system (primary, secondary, and tertiary). The primary level comprises Community-based Health Planning and Services (CHPS) compounds, clinics, health centres and district hospitals. The secondary level has regional hospitals to which referrals are made from the primary level, and where selected specialized services are offered. The tertiary level consists of hospitals that offer highly specialized and advanced care [15]. The study site, Suntreso Government Hospital, is a district hospital and serves Bantama sub-metro district in Kumasi (Ghana's second largest city) and other adjacent districts. The hospital has a staff capacity of 336 which includes doctors, nurses, technicians, administrators, and other auxiliary staff. It has 90 beds and offers a wide range of clinical services. A cross-sectional design was used. We estimated a sample size of 196 which would give a point estimate with a 95% confidence interval of +/- 7%. To account for 15% non-response, we aimed to approach approximately 250 health professionals.

**Study population:** the participants eligible for the study were HCWs who performed medical and surgical procedures or who otherwise interacted directly with patients in their day-to-day activities. We sought to interview the following occupational groups: doctors, nurses, laboratory technicians, pharmacy staff, health informants, and public health workers. Personnel at the management and administration unit, stores and procurement unit, and registry were excluded from the study. Out of 336 eligible HCWs, we randomly selected 250 to approach for interviews.

**Data collection:** eligible HCWs who consented were verbally administered a questionnaire that asked about occupational injuries that might have occurred during the past year. The questionnaire used both open and closed questions and had four components: demographic characteristics, details of any injuries, working conditions, and use of universal precautions. Data were gathered from June to August 2018. Data were collected using smartphones installed with open data kit software. Each interview lasted 30 - 45 minutes. For purposes of this study, an injury was defined as: cut or prick from a needle or other sharp object, blunt injury from a fall or being hit by an object, or a physical injury or threat of such from violence at the workplace.

**Statistical analysis:** data on demographic characteristics, types of injuries, frequency of risk factors, and availability of safety programs were described with proportions and means and standard deviations. Proportion of HCWs reporting at least one injury during the recall year was calculated. Incidence rate of injuries was calculated per person year of exposure based on the denominator of time worked at the hospital during the prior year. Differences in proportions of HCWs reporting an injury by different variables (demographic characteristics, safety practices, and risk factors) were tested using univariable logistic regression analysis. Variables associated with injury at the p<0.20 level were included in a multivariable model to test independent association with risk of injury.

**Ethical considerations:** informed consent was obtained from each participant. Data collection was anonymous. The study was approved by the Committee on Human Research and Publication Ethics of the Kwame Nkrumah University of Science and Technology (CHPRE/AP/378/18).

## Results

**Background characteristics:** out of 250 HCWs selected, 246 could be contacted and consented to be interviewed, for a response rate of 98.4%. The participants were mostly female, aged 25 - 34 years, and married (Table 1). Most were nurses and had either diplomas or first degrees. They had a wide range of work experiences, with a mean of 6.1 years. The distribution of work experiences was: less than a year (34.2%), 1-5 years (40.2%), 6-10 years (14.5%), and more than 10 years (11.1%).

**Table 1:** socio-demographic characteristics of the study sample

Variable	Frequency	Percentage
**Age**		
Less than 25 years	86	35.0
25-34 years	128	52.0
35 year and above	32	13.0
Mean age (SD)	27.76 (6.7)	-
**Gender**		
Male	64	26.0
Female	182	74.0
**Marital status**		
Married	93	37.8
Single	153	62.2
**Profession**		
Physician	23	9.4
Laboratory scientist	14	5.7
Nurse	191	78.0
Other non-clinical staff (health informants, public health workers)	17	6.9
**Religion**		
Christian	231	93.9
Muslim	15	6.1
**Ethnicity**		
Akan	211	85.8
Other ethnic groups	35	14.2
**Educational level**		
Diploma	104	42.6
First degree	137	56.2
Post-graduate	3	1.2

Missing data: profession (1)

**Occupational health practices:** most respondents indicated that they had received training on occupational health and safety (Table 2). Most indicated that they did adhere to safety practices such as properly disposing of sharps and universal precautions. However, there were notable deficiencies in knowledge about specific resources at the workplace. Very few (5.3%) knew who the officer-in-charge was for post-exposure prophylaxis and few (26.4%) knew that there was an occupational health and safety unit at the hospital.

**Table 2:** occupational health practices

Variables	Frequency	Percentage
**Trained in occupational health & safety**		
No	93	37.8
Yes	153	62.2
**Adherence to infection control techniques**		
No	20	8.1
Yes	226	91.9
**Do you properly dispose of sharps**		
No	33	13.4
Yes	213	86.6
**Do you use personal protective equipment (PPE)**		
No	35	14.2
Yes	211	85.8

**Risk factors for injury:** participants reported a variety of risk factors, including a preponderance of shift work (with time of work varying) with few fixed breaks (Table 3). Most reported that their work was stressful. For those 180 respondents reporting stress, the causes were: excessive workload (48.9%), inadequate staff (35.6%), and long working hours (15.6%). They also reported a variety of musculoskeletal stressors, including prolonged standing and moving heavy objects, including patients.

**Table 3:** risk factors for occupational injuries among the health care workers

Variables	Frequency	Percentage
**Stress at work**		
No	66	26.8
Yes	180	73.2
**Working hours**		
1-6 hours	98	39.8
7-8 hours	134	54.5
Above 9 hours	14	5.7
**Fixed breaks**		
No	227	92.3
Yes	19	7.7
**Nature of work schedule**		
Fixed working hours	14	5.7
Run shifts	246	94.3
**Moving heavy objects including patients**		
No	37	15.0
Yes	209	85.0

**Injuries and their outcomes:** seventy-two participants reported an injury within the past year, for a 12-month prevalence of 29.7%. The participants reported a total of 318 injuries, for an incidence rate of 1.63 injuries per person-year of exposure. The most common injuries were needlesticks (27.4% of 318 injuries) and other sharps injuries (26.7%). Also common were blunt injuries due to being hit by objects (19.5%), injuries from workplace violence, (18.9%), and falls (7.5%). The objects involved in “hit by object” included boxes of instruments, oxygen cylinders, infusion stands, among others. Taken together, there were a total of 172 needlesticks and other sharps injuries for an incidence rate of 0.88 per person-year. Of the 72 people who had at least one injury in the past year, most indicated that they reported the injury (Table 4). Most did require treatment for at least one of their injuries, but in all except one case they were treated as outpatients. Approximately half required days off from work as a result of at least one of their injuries. A substantial number (20.8%) reported that they had trouble seeking care due to factors such as the absence of post-exposure medication.

**Table 4:** injury outcome and treatment among healthcare workers at the facility for those people having at least one injury during the past year (n=72)

Variables	Frequency	Percentage
**Was the injury reported**		
No	32	44.4
Yes	40	55.6
**Injury management outcome**		
No treatment	28	38.9
Treated and discharge same day	43	59.7
Hospitalized 1-3 days	1	1.4
**Off days as a result of injury**		
No	37	51.4
Yes	35	48.6
**Thought of changing profession due to injury**		
No	61	84.7
Yes	5	6.9
Maybe	6	8.3
**Difficulty in seeking care**		
No	57	79.2
Yes	15	20.8
Main difficulty faced in seeking care*		
Absence of injury protocol	6	40.0
Absence of occupational health therapist	3	20.0
Absence of post-exposure medication/remedy	6	40.0

* For those 15 people reporting a difficulty in seeking care

**Associations with risk of injury:** the association of injury with the demographic factors and other potential risk factors considered in Table 1, Table 2, Table 3 was evaluated. Four variables had association with injury at the p<0.20 level on univariable analysis (Table 5, Table 5 suite) and were included in the multivariable model. Education level (Table 1) was not included due to colinearity with profession, which was included. Two variables were independently associated in the multivariable analysis. Stress at work was associated with increased risk of injury: aOR 2.68 (95% CI 1.26, 5.71); being a laboratory worker was associated with increased risk of injury: aOR 3.26 (95% CI 1.02, 10.50).

**Table 5:** univariable and multivariable analysis of correlates of occupational injuries among health workers

	Univariable logistic regression		Multivariable logistic regression*	
Variables	Crude odds ratio (OR; 95% CI)	P-value	Adjusted odds ratio (AOR; 95% CI)	P-value
**Age**				
> 35 year (ref)	1			
25-34years	1.51(0.62-3.65)	0.35		
Less than 24 years	0.96(0.37-2.47)	0.94		-
**Gender**				
Male (ref)	1			
Female	0.97(0.52-1.81)	0.93		-
**Marital status**				
Single (ref)	1			
Married	1.61 (0.92-2.81)	0.09	1.36 (0.75 - 2.45)	0.31
**Religion**				
Christian	1			
Muslim	2.98(1.03-8.56)	0.04	2.08 (0.69 - 6.27)	0.20
**Ethnicity**				
Akan	1			
Other ethnic groups	1.52(0.72-3.21)	0.27		-
**Profession**				
Other non-clinical staff (ref)	1			
Nurse	1.88(0.52-6.82)	0.33		
Physician	2.04(0.44-9.43)	0.36		
Lab	4.66(0.91-23.79)	0.06	3.26 (1.02 - 10.50)*	0.047
**Working hours**				
Above 9 hours	1			
7-8 hours	1.02(0.30-3.46)	0.96		-
1-6 hours	1.05(0.30-3.62)	0.93		-
**Stress at work**				
No	1			
Yes	2.56 (1.25-5.25)	0.01	2.68 (1.26 - 5.71)	0.011
**Fixed breaks**				
No	1			
Yes	0.85(0.29-2.46)	0.76		

**Table 5(suite):** univariable and multivariable analysis of correlates of occupational injuries among health workers

	Univariable logistic regression		Multivariable logistic regression	
	Crude Odd ratio (OR; 95% C.I)	P-value	Adjusted Odd ratio (AOR; 95% CI)	P-value
Nature of work schedule				
Fixed working hours	1			
Run shifts	2.5(0.56-11.88)	0.22		-
Moving heavy objects including patients				
No	1			
Yes	1.93(0.80-4.46)	0.34		-
Trained in occupational health and safety				
No	1			
Yes	1.20(0.68-2.13)	0.52		-
Safety protocol displayed				
No	1			
Yes	1.06(0.55-2.02)	0.85		-

* In multivariable model, comparison is to all other professions

## Discussion

We sought to estimate the rate of occupational injuries among HCWs at a typical government, district hospital in Ghana, to assess the availability of safety measures, and to assess risk factors for injuries. We found that the 12-month prevalence rate for having at least one injury was 29.3% and that the annual incidence rate of injuries was 1.63 injuries per person-year of exposure. The most common injuries were needlesticks and other sharps injuries. Also common were blunt injuries due to being hit by objects and injuries from workplace violence. Most respondents indicated that they had received training on safety and followed common safety practices, such as sharps disposal. However very few knew who the responsible officer was for post-exposure prophylaxis or that there was an occupational health and safety unit at the hospital. This study adds to the modest existing literature on health and safety for HCWs in Ghana and Africa more widely. The existing literature has focussed primarily on sharps related injuries and blood-borne exposures and especially on hepatitis B [6,8-10,12,16-19]. An older study (2005) estimated the rates of needlestick and sharps injuries in different regions of the world. Injuries per HCW were logarithmically higher in LMICs (e.g. Africa with 2.1 injuries per HCW per year; Latin America with 2.5; India with 2.3) compared with high-income countries (e.g. Western Europe with 0.64; USA with 0.18) [20]. The current study corroborates that such emphasis is warranted, as needlesticks and sharps injuries did account for the majority of injury mechanisms. It is interesting to note that the annual rate of needlestick and sharps injuries in the current study was 0.88 per HCW per year, less than half of the rate reported for Africa in the above 2005 study. This might be due to recent efforts globally and in Ghana to promote protection against blood-borne infections for HCWs. The rate is still substantially higher than the 0.18 reported for the USA, so there is still work to be done.

The existing literature on HCW safety in Africa and Ghana shows generally low levels of knowledge of universal precautions. For example, one study from rural Ghana reported that only 37% of HCWs knew that standard precautions included hand washing before and after patient contact [17]. Similarly, a study of eight LMICs showed widespread shortages of personal protective equipment needed to protect against blood-borne disease exposure. Interestingly, Ghana was the best supplied for any country studied for the availability of sterile gloves and sterilizers [21]. The current study does show a high degree of awareness of the importance of safe practices, as well as self-reported use of these practices. The differing findings of the current study and the prior study from Ghana may be due to the prior report being from rural hospitals and the current study is urban-based. Although there should not be a difference in standards, it is conceivable that safety practices could vary by urban vs. rural location. The current study also shows that most HCWs felt stress, often related to excessive workload and/or inadequate staffing. A study in Nigeria also showed a high prevalence of work-related stress (83% of all HCWs surveyed) [19]. Increased stress has also been shown to increase exposure to occupational health hazards in hospitals in Uganda [22]. A similar study among 422 HCWs in Ghana showed that work pressure was correlated negatively with safety behavior. However, increased management commitment to safety moderated the relationship between work pressure and safety behavior [23,24].

Also common in the current study were blunt injuries due to a variety of mechanisms, including slips and falls and being hit by objects, and acts of violence. Similar types of injuries have been reported from Uganda and Nigeria [19,22]. The overall topic of violence against HCWs has received considerable attention in some high-income countries and in China [25,26]. It is only beginning to be addressed in sub-Saharan Africa, with just a few studies in the literature, including from South Africa and Botswana [27,28]. Before concluding, the study limitations must be addressed. The frequency of injuries and the use of safety practices and other associated factors were based on self-report by respondents. There was no way to independently validate the veracity of the responses, especially as many did not report their injuries. There is likely under-reporting of injury incidents due to recall bias. Likewise, there is a possibility of over-reporting of good safety behaviors, as respondents might be inclined to give the socially-acceptable answers. Second, due to the time limitation of the HCWs, more in-depth research techniques such as focus group discussions, key informant interviews, and observation of the work process could not be done, which could have given a truer picture of the occupational health situation. Finally, these data are from one hospital and may not be generalizable to the rest of the country. Despite these limitations, this study has the strengths of having a high-response rate and having interviewed a substantial number of HCWs of diverse backgrounds. The study provides us with useful information on ways to improve occupational safety for HCWs in Ghana and in LMICs more widely.

## Conclusion

There is an unacceptably high rate of occupational injuries to HCWs at this hospital in Ghana. This is especially true for needlesticks and sharps injuries that have the potential to spread blood-borne infections. In attempting to address the problem of occupational injuries to HCWs, we have a solid foundation to build on. Most of the HCWs surveyed had prior training about occupational safety and reported high levels of safety-related behaviors. Expanding on this foundation could be done at low cost by making HCWs better aware of existing resources and by removing impediments to care for injuries when they occur, such as increasing capability for post-exposure prophylaxis. For this, the hospital´s office of occupational health and safety needs to be strengthened and expanded. Additional problems, such as workplace violence, need to be considered. Surveillance and reporting need to be strengthened, as many HCWs did not report their injury.

### What is known about this topic

- Injuries to health care workers are more common in low- and middle-income countries than in high-income countries;
- Injuries due to needlesticks and blood-borne exposures have been moderately well studied in low- and middle-income countries and in Africa, but overall occupational safety for health care workers has been less well studied.

### What this study adds

- This study shows the prevalence of all mechanisms of injury to health care workers, including needlesticks, cuts from sharp objects, and violence;
- This study estimates overall injuries and needlestick/sharp injuries per person-year;
- Most workers had training on occupational safety, but there are specific gaps to address, especially for post-exposure prophylaxis.
